# eMental Health Experiences and Expectations: A Survey of Youths' Web-Based Resource Preferences in Canada

**DOI:** 10.2196/jmir.3526

**Published:** 2014-12-17

**Authors:** Felicia M Wetterlin, Marissa Y Mar, Erika K Neilson, Gregory R Werker, Michael Krausz

**Affiliations:** ^1^Institute of Mental HealthDepartment of PsychiatryUniversity of British ColumbiaVancouver, BCCanada; ^2^Centre for Health Evaluation and Outcome SciencesUniversity of British ColumbiaVancouver, BCCanada; ^3^Sauder School of BusinessUniversity of British ColumbiaVancouver, BCCanada; ^4^School of Population and Public HealthUniversity of British ColumbiaVancouver, BCCanada

**Keywords:** mental health services, online systems, adolescents, survey, Internet

## Abstract

**Background:**

Due to the high prevalence of psychological disorders and the lack of access to care among Canadian youth, the development of accessible services is increasingly important. eMental Health is an expanding field that may help to meet this need through the provision of mental health care using technology.

**Objective:**

The primary goals of the study are to explore youth experiences with traditional and online mental health resources, and to investigate youth expectations for mental health websites.

**Methods:**

A Web-based survey containing quantitative and qualitative questions was delivered to youth aged 17-24 years. Participants were surveyed to evaluate their use of mental health resources as well as their preferences for various components of a potential mental health website.

**Results:**

A total of 521 surveys were completed. Most participants (61.6%, 321/521) indicated that they had used the Internet to seek information or help for feelings they were experiencing. If they were going through a difficult time, 82.9% (432/521) of participants were either “somewhat likely” or “very likely” to use an information-based website and 76.8% (400/521) reported that they were either “somewhat unlikely” or “very unlikely” to visit social media websites for information or help-seeking purposes during this time. Most (87.7%, 458/521) participants rated their online privacy as very important. Descriptions of interventions and treatments was the most highly rated feature to have in a mental health-related website, with 91.9% (479/521) of participants regarding it as “important” or “very important”. When presented a select list of existing Canadian mental health-related websites, most participants had not accessed any of the sites. Of the few who had, the Canadian Mental Health Association website was the most accessed website (5.8%, 30/521). Other mental health-related websites were accessed by only 10.9% of the participants (57/521).

**Conclusions:**

The findings suggest that despite interest in these tools, current eMental Health resources either do not meet the needs of or are not widely accessed by youth with mental health problems. In order to improve access to these resources for Canadian youth, Web-based platforms should provide information about mental health problems, support for these problems (peer and professional), and information about resources (self-help as well as ability to locate nearby resources), while protecting the privacy of the user. These findings will not only assist in the development of new mental health platforms but may also help improve existing ones.

## Introduction

Canadians are heavy users of the Internet [1]. In 2011, 83% of Canada’s population reported using online services [2], and in 2014, 99% of young Canadians reported having access to the Internet outside of school [3]. The number of individuals using the Internet to search for medical or health-related information increased from 57.9% in 2005 to 69.9% in 2009 [4]. With such a large part of Canada’s population online, and with many young adults using the Internet for health-seeking purposes, it is critical to understand more about the health-seeking behaviors, experiences, and expectations of youth.

eMental Health is an expanding field and is defined by the Mental Health Commission of Canada as “the use of information and communication technologies to improve mental health” [5]. An increased use of the Internet in everyday life has contributed to a rise in self-help and Web-based therapies for various physical and psychological disorders [6,7].

Developing more accessible services has become increasingly important due to the high prevalence of mental disorders—particularly mood, substance use, and anxiety disorders—in Canada. According to the Canadian Mental Health Survey, 20% of Canadians will experience a mental illness during their lifetime, 11.3% will be diagnosed with major depressive disorder, and 8.7% with an anxiety disorder [8]. The National Comorbidity survey in the United States reports an even higher lifetime mental disorder prevalence of 44% [9].

Youth between the ages of 15 and 24 years have the highest prevalence of mental disorders, and yet almost one-third of Canadians seeking mental health care report that their needs are unmet or partially unmet [8]. In one study, only 42% of young adults aged 19-24 years with major depressive disorder had accessed any mental health services [10]. Research suggests that youth view current/traditional mental health services negatively, and that future efforts should center on the development of interconnected and empowering services as a means of increasing service use [11]. The Canadian Institutes of Health Research states that “advanced eHealth innovations feature integrated and sophisticated technology that enables interconnectivity at all levels of the health system” and that “eHealth empowers patients and encourages self-management” [12]. These facts suggest the high level of unmet needs can only be addressed by the addition of significant capacity. The need to develop Web-based mental health services is supported by the perceived acceptability of online therapy as a form of treatment [13], as well as its effectiveness in providing support for youth who have family members experiencing mental illness [14].

To guide the conceptualization and development of future effective, attractive, and youth-appropriate Web-based services, we studied current experiences and expectations of online health solutions among youth by exploring the following questions: (1) What sources of online information are young people most likely to use, and what do they find most accurate, when seeking help for mental health issues?, (2) What sources of online information do young people predict they would use if they had a mental health issue?, and (3) What experience do young people have in using mental health resources on the Internet?

## Methods

### Recruitment

The Bell Youth Impact Survey ran from September 2012 to March 2013 and garnered 521 complete responses. Recruitment of youth living in Canada between 17 and 24 years of age was accomplished through a variety of methods. Participants were recruited via targeted Facebook advertisements; Facebook and Twitter pages dedicated specifically to the survey; internal communiqués at the Centre for Student Involvement at the University of British Columbia, Student Communications Services, the Centre for Health Evaluation and Outcome Sciences, the Paid Participants Studies list; and through partner community organizations.

### Survey Procedure

The survey was developed after a review of existing literature concerning youth self-reports of their mental health and resource use. Questions were created in consultation with a licensed psychiatrist. The survey was then administered to members of the research team as well as non-affiliated individuals for pre-testing before use. Approval for the survey was obtained from the Behavioral Research Ethics Board at the University of British Columbia. All participants, regardless of recruitment source, were directed to a website providing a brief description of the survey. From this website, youth interested in participating were prompted to email the researcher in order to receive a website link to the survey and a unique, single-use access code. Participants who followed the link were presented with a consent form and upon acceptance were redirected to the survey, which was hosted on FluidSurveys.

The first component of the survey consisted of the exclusion criteria. Any participant indicating that they were 16 years or younger, 25 years or older, or not currently living in Canada was redirected to the survey termination page and notified that they were not eligible to complete the survey.

The survey consisted of approximately 65 questions distributed over a maximum of 16 pages (depending on skip logic), and took between 15 and 20 minutes to complete. Participants were permitted to skip any question they were unwilling to answer and to discontinue the survey at any time. They were also able to review and change their answers during the course of the survey. Participants who completed the survey and provided an email address received a CAN $5 gift card to a retail store of their choice (Starbucks, Chapters, or Amazon).

Only completed questionnaires, in which all pages were visited (even if some were skipped), were included in the analysis. Participant identifiers were removed from the survey before data analysis.

### Measures

The survey consisted of both quantitative and qualitative questions to assess: (1) demographics, (2) mental health literacy, (3) online and offline use of mental health resources, (4) online and offline opinions about current mental health resources, and (5) preferences regarding potential components of a mental health website. In this paper, we include items (3), (4), and (5).

## Results

### Participant Demographics

Out of the 521 participants, 76.6% were female (399/521) and the mean age was 20.68 years (SD 2.08). Most participants were East/Southeast Asian (44.0%, 229/521) or European/Caucasian (35.5%, 185/521). Most (61.6%, 321/521) participants indicated that they had used the Internet to seek information or help for the feelings they were experiencing. Participant location, ethnicity, and education status are presented in Table 1.

When asked what they were looking for on the Internet in the context of seeking mental health information (Table 2), 52.4% of participants (273/521) indicated that when using the Internet for mental health information-seeking purposes they were looking for information about symptoms and 47.4% (247/521) were looking for treatment options. A total of 2.5% participants (13/521) selected “other” as a response and provided their own answer, with common themes such as looking for personal testimonies from people with the same mental health problem, coping mechanisms for their family, counseling or crisis chats, self-help strategies, and ways in which to cope.

**Table 1:** Participant demographics (n=521).

Demographic		n (%)
**Gender**		
	Male	121 (23.2)
	Female	399 (76.6)
**Current location**		
	British Columbia	448 (86.0)
	Ontario	43 (8.3)
	Newfoundland	10 (1.9)
	Nova Scotia	6 (1.2)
	Alberta	3 (0.6)
	Manitoba	3 (0.6)
	Quebec	3 (0.6)
	Prince Edward Island	1 (0.2)
	Yukon	1 (0.2)
**Student status**		
	Currently in school (eg, high school or post-secondary education)	455 (87.3)
	Not currently in school	65 (12.5)
**Ethnicity**		
	East/Southeast Asian	229 (44.0)
	European/Caucasian	185 (35.5)
	South Asian	63 (12.2)
	West Asian	32 (6.1)
	Latin/Central/South American	15 (2.9)
	Aboriginal	8 (1.5)
	Caribbean	7 (1.3)
	Arab	5 (1.0)
	African	5 (1.0)
	Pacific Islander	3 (0.6)

**Table 2:** Web-based mental health information-seeking tendencies (n=521).

Features	n (%)
Information about symptoms	273 (52.4)
Information about treatment options	247 (47.4)
Information about prevalence rates	90 (17.3)
Web-based questionnaires or assessment tests	124 (23.8)
Peer support	82 (15.7)
A list of local resources	68 (13.1)
Other	13 (2.5)

### Resource Use

**Figure 1:**
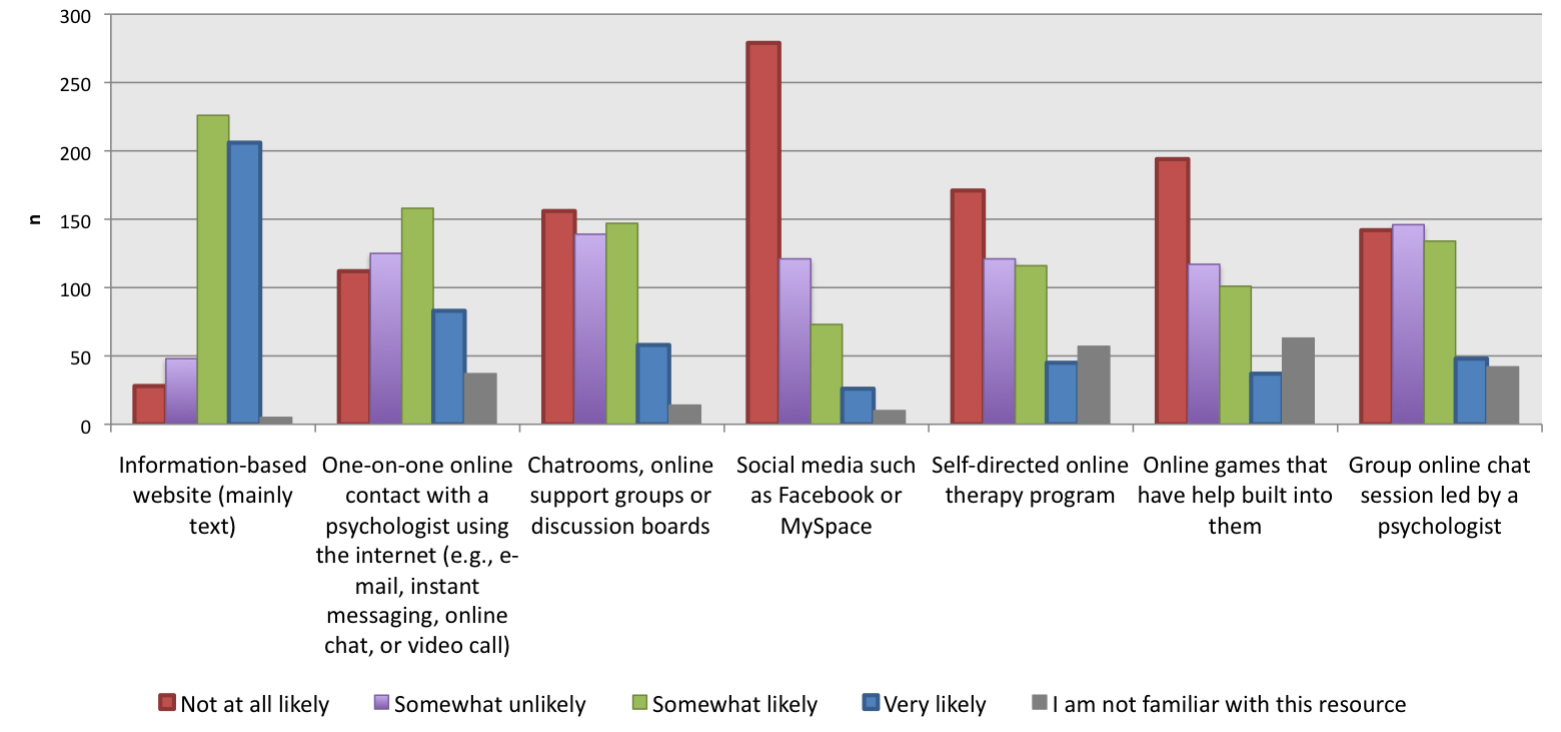
Likelihood of visiting Web-based resources during a difficult time in life.

When asked about the likelihood of visiting certain types of Web-based resources during a difficult time in life (Figure 1), most (82.9%, 432/521) participants were either “somewhat likely” or “very likely” to use an information-based website with mainly text. Slightly more than half of the participants were “not at all likely” or “somewhat unlikely” to use online interactions such as a group online chat session led by a psychologist (55.3%, 288/521) and chat rooms/support groups/discussion boards (56.6%, 295/521). More than half (59.7%, 311/521) of participants stated that they were “not at all likely” or “somewhat unlikely” to use online games that have help built into them. The majority (76.8%, 400/521) reported that they were either “somewhat unlikely” or “very unlikely” to visit social media websites during this time.

**Figure 2:**
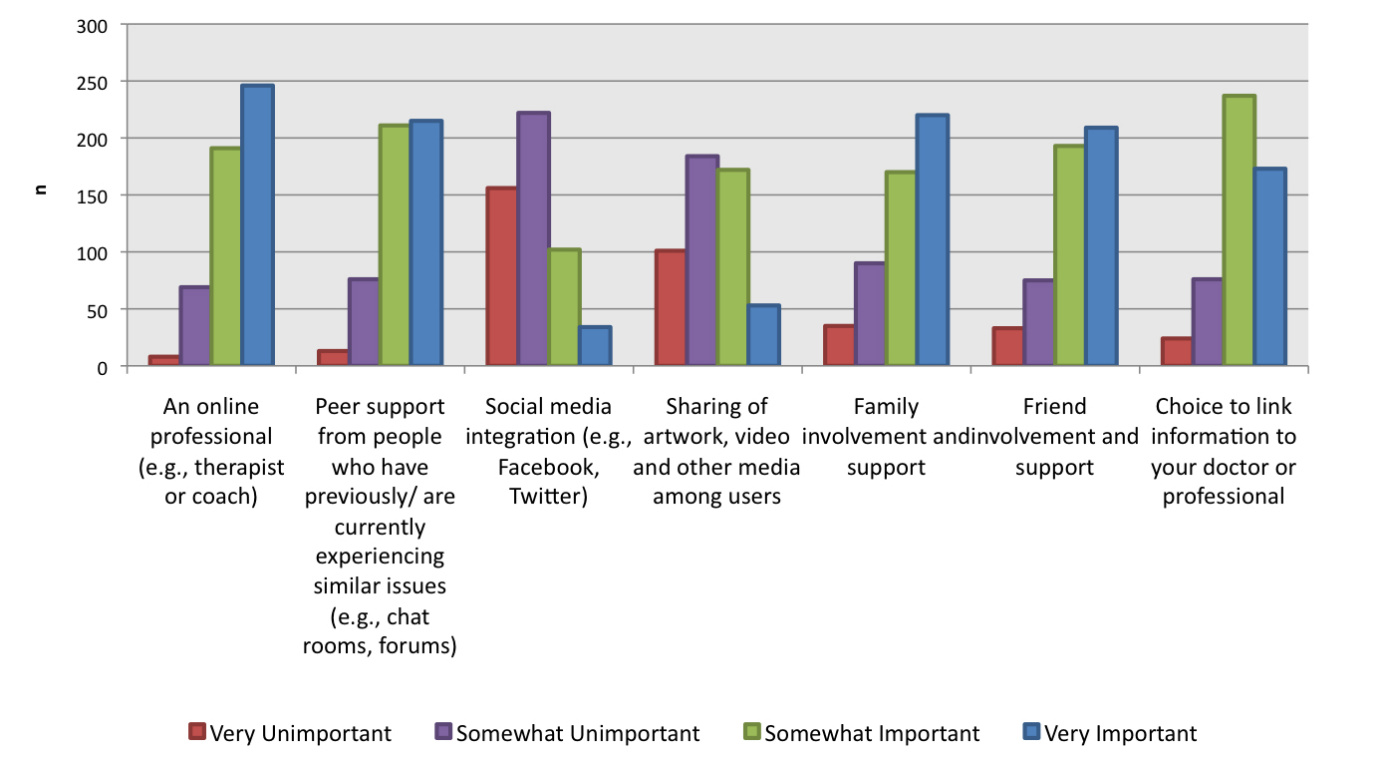
Importance of contact with different supports in a Web-based mental health resource.

When asked to rate the importance of human contact within a Web-based mental health resource (Figure 2), the majority of participants (83.9%, 437/521) listed contact with an online professional (eg, therapist or coach) as either somewhat important or very important. Peer support (81.8%, 426/521), family involvement (74.8%, 390/521), and friend involvement (77.1%, 402/521) were other popular responses listed as “somewhat important” or “very important” as contacts to have within a Web-based mental health resource.

Out of the 322 participants who indicated that they had used the Internet to look for information or help about what they were feeling, only 10.6% (34/321) said that they had used social media (eg, Facebook, MySpace) to obtain help with problems such as anxiety or depression.

**Figure 3:**
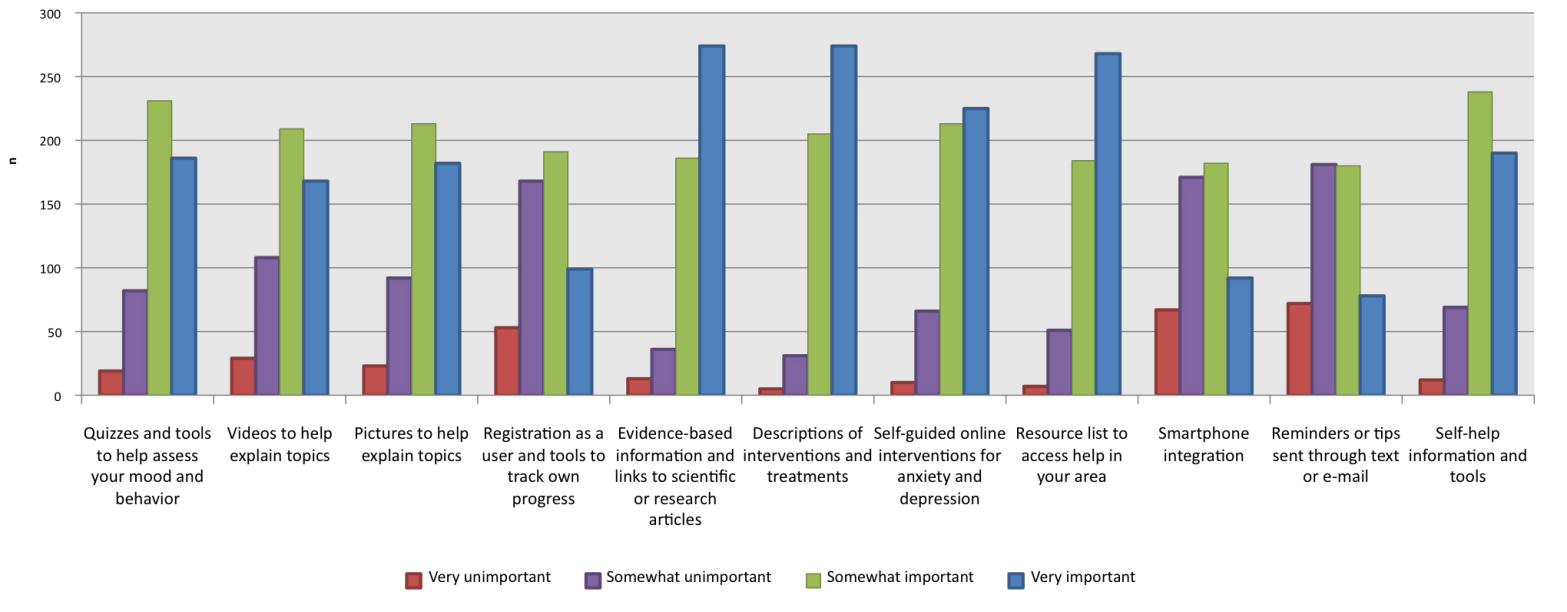
Importance of different features within a Web-based mental health resource.

Figure 3 shows the values and corresponding percentages of the responses given when participants were asked to rate the importance of certain features as components of a Web-based mental health resource. The most highly rated features (regarded as “important” or “very important”) by participants were descriptions of interventions and treatments (91.9%, 479/521), evidence-based information and links to scientific or research articles (88.3%, 460/521), a resource list to access help “in your area” (86.7%, 452/521), self-guided Web-based interventions for anxiety and depression (84.1%, 438/521), self-help information and tools (82.2%, 428/521), quizzes and tools to help assess mood and behavior (80.0%, 417/521), pictures to help explain topics (75.8%, 395/521), and videos to help explain topics (72.3%, 377/521).

### Privacy

Most participants (87.7%, 458/521) rated their privacy as a user as “very important”.

### Website Familiarity

Participant familiarity with current popular mental health-related websites was assessed by asking participants to indicate, from a list of Canadian websites related to mental health and well-being, which they had visited. Most participants had not visited any of the websites, but, of the minority who had, the Canadian Mental Health Association website was the most highly accessed Canadian mental health-related website (5.8%, 30/521). The remaining websites were only accessed by 10.9% of the participants (57/521).

## Discussion

### Principal Findings

With 93% of teens and young adults regularly online [15], Web-based mental health resources need to become a priority. Easy access to information and treatment options about mental health disorders can better facilitate early detection and treatment of psychological disorders in youth. Our findings show that the Internet is a key resource for youth between the ages of 17 and 24 years when searching for information on symptoms or when looking for help concerning their mental health. This focus on Web-based resources may be linked to the inadequacy of offline/traditional mental health services in providing information and services for all who seek it [16].

Participants rated the importance of online support in the form of online contact with a psychologist or mental health professional, peer support, friend involvement, and family involvement as important features of a mental health-related website. This finding may suggest an underlying desire to seek human interaction as a part of an online community without interfering with daily life or compromising privacy.

However, when asked to rate their personal likelihood of using services designed to provide support (such as group online chats with a psychologist, chat rooms, and support groups), the majority of participants stated that they were unlikely to use these services. This discrepancy in results between Figures 1 and 2 may be influenced by our sample, which included both those with mental health issues and those without. For example, participants without mental health problems may have underestimated what features they would use if they were going through a difficult time in their life. Alternatively, this difference in responses may be explained by the psychological phenomenon—pluralistic ignorance—in which people tend to collectively assume that their opinion of a norm or belief is the minority and that the majority thinks differently than they do [17]. In the words of Krech and Crutchfield, “no one believes, but everyone thinks that everyone believes” [18]. In this instance, the average person did not personally need online support, but believes that others do. If this is the case, the response rate of Figure 2 may be an overestimate of the importance of contact with different supports in Web-based mental health resources for youth. Other research has found similar trends in hypothetical information-seeking and actual usage of Web-based sources, with a higher percentage of participants indicating that they were likely to use a certain feature than the percentage of participants who had actually used the feature [19].

Youth do not appear to use social networking sites when looking for help or support for problems such as anxiety or depression. Most participants rated their privacy on the Internet as very important, which may explain the lack of social networking site use for mental health help or support. This avoidance of social networking sites may also be due to the stigma associated with mental illness [20] and the public nature of such sites. Because of the reported tendency for youth to not use social networks for mental health concerns, it may be important for Web-based platforms to discuss mental health problems in a way that fosters a sense of community while ensuring anonymity for the user. This could help youth with mental health problems connect with similar peers in a safe environment.

Most features (Figure 3) in the survey were rated as being important components of a mental health-related website, suggesting that a variety of features are likely to meet the needs of the youth population. A mental health website would need to have information from credible and trustworthy sources, including descriptions of interventions and treatments. This finding is consistent with previous research [21], which suggests that the current adolescent generation faces the challenge of finding reliable information online.

Of the currently available mental health-related websites we asked about, very few of the participants had accessed any at all. Reasons for this lack of access are unclear. It may be due to lack of awareness of existing mental health-related websites as a result of advertising (ie, perhaps these websites are helpful, but are not advertised and therefore not recognized or accessed by participants), or perhaps they are inadequate and there are no sites that meet users’ needs. Alternatively, in our study, we may have inquired about the “wrong” websites (ie, websites that do not provide the needed services and are therefore not used by young people searching for mental health resources) but other sites are used and seen to be useful. This finding is supported by the systematic review by Krauer et al [22], which analyzed help-seeking behavior among young people and found that current Web-based mental health resources do not contribute significantly to help-seeking behaviors. The review, however, does state that young people are inclined to use Web-based resources. Rickwood et al have further explored the subject in a review that underlined the need for mental-health help to be easily accessible and youth-friendly [23]. Help-seeking behavior in youth might therefore be enhanced by aligning Web-based mental health resources with the preferences of youth as identified in this paper.

### Limitations

The findings of this study are limited to youth between the ages of 17-24 years living in Canada, the majority of whom are pursuing a post-secondary education. Females are more likely to seek out and engage with services, so our results may not generalize to the Canadian youth population as a whole [23]. Most participants were living in British Columbia and reflected the general make-up of the province [24], and it is reasonable to expect similar preferences from youth across different provinces. Although the sample largely consisted of European/Caucasian and East/South East Asian youth, it should be noted that the survey was designed in such a way that participants could identify as more than one ethnicity. Therefore, any generalizations between ethnicity and Web-based mental health service use and expectations should be made with caution. Another limitation is that the study relied on self-reported data to determine eMental Health resource use and the expectations of future Web-based resources. Because some of the questions allowed the participants to select more than one response, it was not possible to determine rank of choices and therefore popularity of the features relative to one another. Last, this survey also asked participants about hypothetical situations, emotions, and Web-based mental health resources, which may not be reflective of actual behavior.

### Conclusions

These findings suggest that the next step in Web-based mental health care is to ensure that online platforms (not social media-based) provide information about mental health problems, support for these problems (peer and professional), and resources (self-help as well as information on nearby resources), while protecting the privacy of the user. These findings will not only assist in the development of new mental health platforms, they may also help improve existing ones.
